# Numerical Simulation of the Influence of Geometric Configurations on Pressure Difference in the Intraventricular Tunnel

**DOI:** 10.3389/fphys.2020.00133

**Published:** 2020-02-21

**Authors:** Yao Yang, Junjie Wang, Aike Qiao, Xiangming Fan

**Affiliations:** ^1^Beijing Anzhen Hospital, Capital Medical University, Beijing, China; ^2^College of Life Sciences and Bioengineering, Beijing University of Technology, Beijing, China

**Keywords:** double outlet right ventricle, intraventricular tunnel, hemodynamics, numerical simulation, surgical planning

## Abstract

**Objective:**

The geometric configuration of the intraventricular tunnel is related to the re-intervention of left ventricular outflow tract stenosis after double outlet right ventricle (DORV) correction. Hemodynamic simulation was performed in order to study the influence of the geometric configuration of the IVT on the pressure difference.

**Methods:**

CT images of DORV were processed to reconstruct 3D models of left and right ventricular flow chambers and aortic valve orifice, and then the size and relative position of the aortic valve orifice and ventricular septal defect were determined. Twenty five groups of the idealized models were established according to orthogonal test design and computational fluid dynamics method was applied to simulate hemodynamics. Three factors of geometric configuration were considered for the study of their influences on the pressure difference. The first factor is the distance between the ventricular septal defect and the plane of the aortic valve (D_SA_), the second factor is the ejection angle of blood from left ventricle flowing into the IVT (A_LT_), and the third factor is the turning radius of the IVT (R_TT_). SPSS software was employed to perform the orthogonal analysis. Additionally, twelve models with different turning radii were established for hemodynamic analysis, with the turning radii increasing from 0 mm with an interval of 1 mm, so as to study the influence of turning radius on pressure difference of IVT.

**Results:**

The analysis of variance showed that only the change of R_TT_ had a significant effect on the pressure difference (*P* = 0 < 0.05), while the change of D_SA_ and A_LT_ had no significant effect on the pressure difference (*P* = 0.459 > 0.05, *P* = 0.263 > 0.05). The pressure difference decreases with the increase of R_TT_. When R_TT_ reaches 6 mm, the pressure difference gradually remains unchanged with the increase of R_TT_, and the rate of change is less than 5%.

**Conclusion:**

R_TT_ in the IVT is the main factor affecting the pressure difference. A small R_TT_ will lead to a large pressure difference in the IVT. When R_TT_ increases to 6 mm, the pressure difference in the IVT remains nearly unchanged. When performing the right ventricular double outlet correction; the turning radius of the IVT should be about 6 mm to ensure relatively small pressure difference.

## Introduction

Double outlet right ventricle (DORV) has been a contentious topic in congenital heart pathology and surgery. In 2000, the International Association of Thoracic Surgeons and the European Association for Cardio-Thoracic Surgery adopted a new naming rule for DORV, namely the “50%” rule: one aorta and more than 50% of the other originate in the right ventricle and the only outlet of the left ventricle is the ventricular septal defect (VSD) (Lacour-Gayet et al., 2002). VSD is one of the most common cardiovascular malformation, referring to disrupt the integrity of tissue between left and right ventricle and result in existing abnormal channel between the ventricles.

Congenital (VSD), which accounts for about 12 to 20% of the congenital cardiovascular malformation, is caused by the development of fetal original ventricular septal dysplasia. It can exist as a single malformation or as part of other complex cardiac malformations, such as tetralogy of fallot, complete atrioventricular malformation, transposition of the great arteries, tricuspid atresia, and double outlet of the right ventricle. The location, size and shape of aortic valve orifice and VSD are the key to preoperative preparation (Villemain et al., 2016).

By combining cardiac CT angiography with 3D printing technology, scholars can accurately determine the pathological classification of DORV before surgery. Pathological heart model analysis and simulated surgery can significantly reduce the intraoperative exploration time of DORV with VSD far from the two main arteries (Lefort et al., 2001; Meduri et al., 2007; Garekar et al., 2016; Pang et al., 2017; Wang et al., 2017; Mostefa-Kara et al., 2018).

Double outlet right ventricle is primarily treated with a surgical treatment, which establishes intraventricular tunnel (IVT) connecting the VSD to the aortic valve orifice to restore normal cardiac function. The 10-year survival rate was 87% and the re-intervention rate was 24% (Lefort et al., 2001). The main post-operative diagnosis is echocardiography, and the pressure difference (ΔP) is the main criterion for evaluating IVT stenosis. Left ventricular outflow tract obstruction occurs when ΔP exceeds 10 mmHg (Ware and Matthay, 2000; Hashirnoto et al., 2004). The most significant re-intervention factors were related to IVT, such as post-operative residual fistula and left ventricular outflow tract obstructions (Marshall et al., 2000; Shoda et al., 2007). IVT stenosis is the most common complication and indication of reintervention (Iwai et al., 2007; Pang et al., 2015; Egbe et al., 2016; Cui et al., 2019).

However, the state-of-the-art studies mainly focus on the anatomical morphology and preoperative diagnosis of DORV, without pointing out the effect of IVT morphology on the left ventricular outflow tract or the suggestions on how to improve IVT morphology from the view point of biomechanics.

The main purpose of this study is to investigate the effect of geometric configuration of IVT on ΔP, and to provide reasonable suggestions and theoretical basis for surgeons to establish IVT.

## Materials and Methods

In order to simplify the calculation, all the models established in this study are idealized models. The size of the idealized model was established according to the 3D reconstruction model of the patient’s ventricle. The diameter of VSD is not equal to the diameter of aortic valve orifice. During the operation, VSD will be cut to make the diameter of VSD equal to that of aortic valve orifice. Therefore, the diameter of the aortic valve in the ideal model established in this study is equal to that of the VSD. Orthogonal test design was used to arrange the experiment and the significance of the experimental results was analyzed. A single factor test was carried out for the factors that had significant influence on the results.

### Model Reconstruction

Orthogonal experimental design is a method to study multiple factors and levels. According to the orthogonality, some representative points are selected from the overall test to carry out the test. These representative points have the characteristics of “uniform dispersion and uniform comparison.” Orthogonal test design is the main method of fractional factorial design, which is an efficient, fast and economical test design method. Therefore, this paper adopts the orthogonal experiment design to arrange experiments, which can not only reduce the number of experiments but also obtain the specific influence factor *P* value of each factor on the results.

**FIGURE 1:**
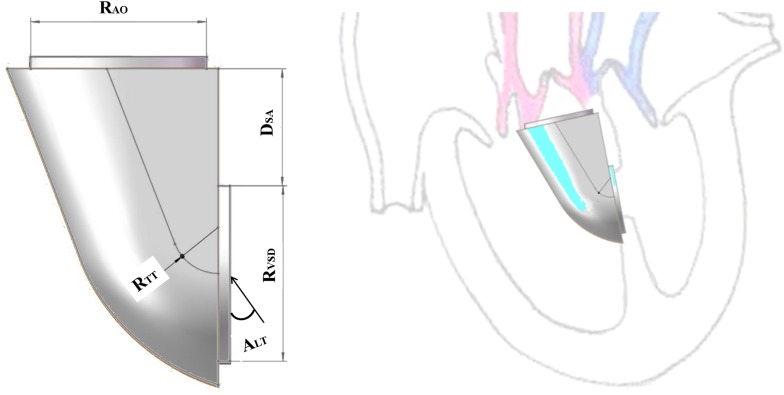
**(A)** Idealized IVT geometry; **(B)**. IVT assembly diagram.

The orthogonal test method was used to arrange the experiment and analyze the results in SPSS. The main factors were the distance (D_SA_), the ejection angle (A_LT_) and the turning radius of the IVT (R_TT_). D_SA_ was the distance between the VSD and the plane of the aortic valve, A_LT_ was the ejection angle of blood from left ventricle flowing into the IVT, and R_TT_ was the turning radius of the IVT (Figure 1). Each factor takes five values. Twenty five groups of the idealized models were established according to the orthogonal test design and computational fluid dynamics (CFD) method was applied to simulate hemodynamics (Table 1).

**TABLE 1:** Orthogonal test design table and results.

Model	D_SA_ (mm)	A_LT_ (°)	R_TT_ (mm)	△P (mmHg)
1	10	20	5	3.27
2	10	10	2.5	5.36
3	15	20	0	6.79
4	20	0	2.5	8.23
5	25	20	2.5	5.6
6	20	40	0	2.88
7	20	20	7.5	3.47
8	25	30	5	2.75
9	30	30	0	8.34
10	15	30	2.5	2.77
11	20	30	10	2.19
12	25	0	10	4.56
13	30	10	7.5	4.43
14	30	20	10	3.25
15	15	40	5	1.49
16	30	40	2.5	1.75
17	10	30	7.5	1.77
18	10	40	10	1.1
19	10	0	0	8.26
20	25	40	7.5	1.23
21	30	0	5	6.6
22	15	10	10	3.89
23	20	10	5	5.13
24	25	10	0	12.91
25	15	0	7.5	5.11

The small steps at the aortic valve and VSD conform to the physiological structure, and there is thickness of the ventricular septum and space below the aortic valve. Therefore, the models established in this paper retain the small steps, which not only simplify the mesh division, but also facilitate the application of boundary conditions.

The results of orthogonal experiment were analyzed with the analysis of variance in SPSS. *P* < 0.05 was considered statistically significant. In this study, only the *P* value of R_TT_ was less than 0.05, so the influence of R_TT_ on ΔP was studied in detail. The R_TT_ was chosen to increase from 0 mm with an interval 1 mm, and 12 groups of models were established. The other sizes of the idealized IVT remained the same.

### Mesh Generation

The idealized models were meshed using Ansys ICEM CFD (Ansys, Inc). Combination of tetrahedral and prism elements were used, with five layers of prism elements along the walls. After grid dependency analysis, the final mesh elements count of IVT was about 150000. CFD analyses of the idealized IVT were conducted using Ansys-CFX.

### Finite Element Analysis

The meshed fluid model was imported into the pretreatment of the finite element analysis software ANSYS-Workbench to set the boundary conditions and blood parameters. The VSD was set as the blood flow inlet, and the aortic root was set as the pressure outlet.

Boundary conditions: Steady-state simulation of ventricular systolic conditions was carried out in this study to mimic the peak flow condition in a cardiac cycle. Although DORV is a congenital heart disease, there was a wide age gap among patients receiving surgical treatment, including infants and adults. Therefore, the selection of boundary conditions in this study was based on the physiological status of normal adults. So the boundary condition of the inlet is set as the flow velocity, 1 m/s, and the boundary condition of the outlet is set as the pressure, 110 mmHg.

Blood parameters: In all numerical simulations of the steady flow of aorta, blood was considered as Newtonian, homogeneous, and incompressible. The fluid satisfies no slip condition on the wall. The fluid viscosity was set to 0.0035 Pa⋅s and the density to 1050 kg/m^3^ [17]. Since the maximum Reynolds number is about 4000, the turbulent models were applied for the hemodynamic simulation.

## Results

According to the variance analysis of the orthogonal experiment results, the influence of the value of each factor on the ΔP could be found: only the change of curvature of the center line has a significant effect on convection resistance (*P* = 0 < 0.05) (Table 2). The distance and the angle have no significant influence on ΔP (*P* = 0.459 > 0.05, *P* = 0.263 > 0.05). Through multiple comparisons of R_TT_, it was found that the increase of R_TT_ would have a significant impact on the results when R_TT_ was small. However, the change of R_TT_ had no significant effect on the result when R_TT_ was larger. For example, it was significantly different from other values when R_TT_ = 0 mm. However, there was no significant difference with R_TT_ = 5 mm and R_TT_ = 10 mm when R_TT_ = 7.5 mm (Table 3).

**TABLE 2:** Anova analysis of orthogonal test.

	Type III sums	The square	*F*	Statistical
	of squares	of average		significance (*P*)
D_SA_	0.004	0.001	1.374	0.459
A_LT_	0.002	0.001	0.63	0.263
R_TT_	64.981	16.245	20477.055	0.000

**TABLE 3:** Multiple comparison single factor.

R_TT_ (mm)		Statistical	R_TT_ (mm)		Statistical
		significance *(P)*			significance (*P*)
0	2.5	0.004	7.5	0	0
	5	0.001		2.5	0.107
	7.5	0		5	0.379
	10	0		10	0.822
2.5	0	0.004	10	0	0
	5	0.332		2.5	0.072
	7.5	0.107		5	0.356
	10	0.072		7.5	0.822
5	0	0.001			
	2.5	0.032			
	7.5	0.379			
	10	0.356			

**FIGURE 2:**
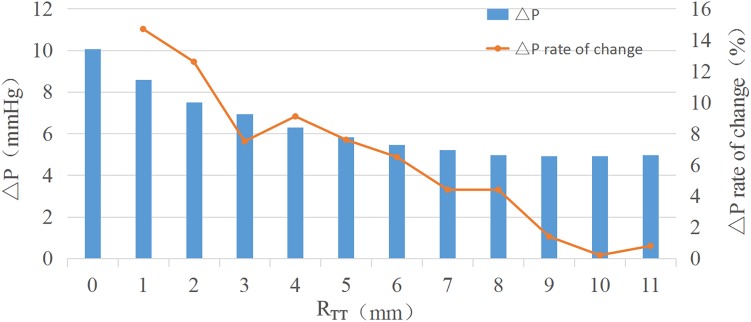
△P-R_TT_ and ΔP rate of change-R_TT._

**FIGURE 3:**
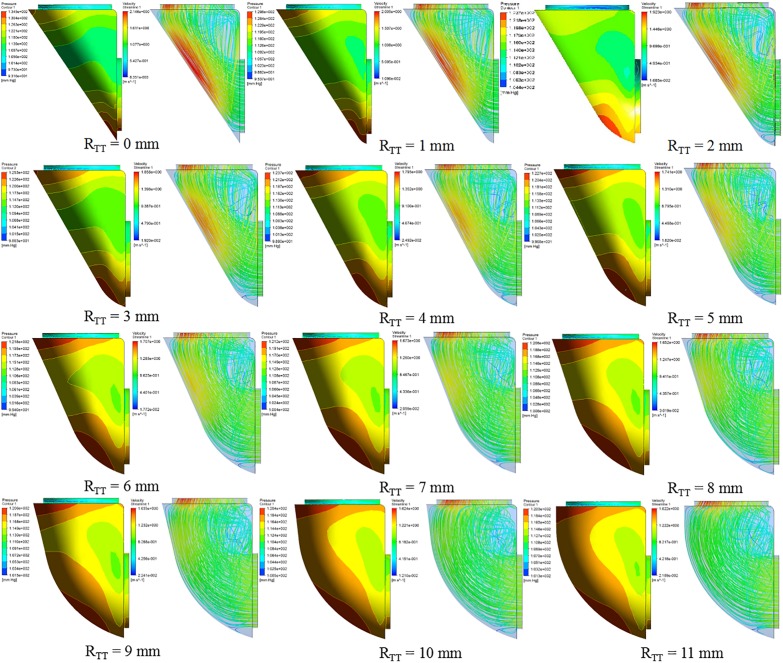
The distribution of pressure and streamline of 12 groups of different R_TT_ models.

Analysis of the orthogonal test results showed that R_TT_ changes had a significant impact on ΔP, so R_TT_ was further studied and ΔP−R_TT_ and ΔP rate of change - relation chart were drawn in Figure 2. The results showed that the ΔP decreases with the decrease of curvature. When the curvature diameter reached 12 mm, the ΔP gradually remained stable with the increase of diameter, and the change rate of ΔP decreased to less than 5%. The IVT internal velocity and wall pressure also decreased with the increase of R_TT_ (Figure 3).

## Discussion

Some studies have demonstrated the effect of IVT morphology on the surgical outcome, however no study to our knowledge has ever included hemodynamic analysis of IVT morphology to investigate the effect of IVT morphology on the ΔP.

The main reason why we adopted the CFD analysis was that computational modeling enabled a quantitative analysis of fluid-dynamic parameters, such as velocity streamlines and pressure, which were impossible to measure non-invasively in clinics with sufficient accuracy. In fact, although transthoracic (TTE) and transoesophageal echocardiography (TEE) are available in the clinic to measure the blood flow velocity in IVT, both techniques can only provide average information on the velocity magnitude and direction in the sampling volume, without being able to give detailed spatial 3D information on the local flow conditions (Fukuda et al., 2003; Dentamaro et al., 2017). Therefore, it is impossible to accurately evaluate the hemodynamics of IVT morphology. Because the inlet velocity and the VSD diameter is constant, the flow rate at the inlet of each model is constant. According to Poiseuille’s law, the mean pressure difference (ΔP) is proportional to the flow resistance when the flow rate is constant. Therefore, the ΔP between the aortic valve and the VSD was used as the evaluation criterion of flow resistance. So the CFD analysis was used to study the influence of IVT morphology on the ΔP.

According to the CFD analysis results of the idealized IVT models, it could be observed that the maximum pressure was concentrated on the IVT wall near the inlet. The high pressure on the IVT was caused by the impact of blood flow on the wall. Under the same model, the position of high pressure area was mainly affected by A_LT_: with the increase of A_LT_, the high pressure area was shifted upward.

According to the conservation of mechanical energy, the increased kinetic energy of fluid is mainly due to the decrease of potential energy. So the increase of the flow rate led to the increase of ΔP. So the increase of velocity in IVT leaded to a sharp increase of ΔP. By comparing CFD results of different R_TT_ models, the increase of R_TT_ could effectively reduce the flow velocity within IVT, and the ΔP also decreased. Anova of orthogonal test results also proved that the change of R_TT_ had a significant impact on the ΔP (*P* < 0.05). Therefore, we could ensure the left ventricular outflow tract unimpeded by changing R_TT_ to make the IVT flow velocity less than 1.2 m/s or ΔP less than 10 mmHg.

As R_TT_ increased, ΔP gradually decreases. When R_TT_ was equal to 6 mm as R_TT_ increased, ΔP largely remained unchanged. An increase in R_TT_ would result in an increase in IVT volume. Since the IVT was established in the right ventricle, the IVT encroached on the volume of the right ventricle. Excessive IVT volume could lead to increased right ventricular pressure, decreased ejection, and ultimately right ventricular dysfunction. The R_TT_ should be 6 mm when constructing the IVT, and the ΔP and volume of the IVT should reach the minimum value.

## Limitations

The main limitations of this study were as follows: (1) The idealized model was adopted for simulation without considering the complex structure of the heart chamber; (2) Only 3paramenters of IVT were selected for research based on experience; (3) CFD applications did not consider fluid-solid interaction (FSI), valves or moving walls, which made it impossible for us to compare any measurements with cardiac functions. However, as mentioned earlier, this study only explored the effect of the IVT geometry sizes on hemodynamics.

Due to the randomness of the geometric size and relative position of aortic valve orifice and VSD in DORV, we selected three factors that may have a greater impact on hemodynamics to establish idealized models for simulation. Therefore, the small sample size of this study limited the generality and statistical significance of the results.

When the blood flow enters the IVT, there was an impact and interaction between the IVT and the blood flow. The blood flow out of the IVT also interacted with the valve. These two interactions would have certain impact on the results, limiting the generality of the results (Töger et al., 2012; Kouchoukos et al., 2013; Vedula et al., 2016), but it was impossible to capture in this study.

All in all, future work will include increasing the geometry sizes of IVT for research, establishing IVT models in real ventricular models and using FSI for simulation. This allows us to compare our findings with *in vivo* measurements.

## Conclusion

The turning radius of the IVT, R_TT_ is the main factor affecting the ΔP. A small R_TT_ will lead to a large ΔP in the IVT. When R_TT_ increases to 6 mm, the ΔP in the IVT remains nearly unchanged. When performing the DORV correction, the turning radius of the IVT should be about 6 mm to ensure relatively low ΔP and small volume.

The CFD model mentioned in this study highlighted the significant influence of IVT shape on ΔP. This computational study supported the hypothesis that IVT shape was the primary cause of DORV surgical complications. Although the method used needs further improvement and large-scale verification, it could quantitatively analyze the parameters that couldn’t be measured in clinical practice and provide theoretical basis for clinical surgery.

## Data Availability Statement

All of the data produced and analyzed in the present study are involved in the manuscript as tables or figures. The corresponding author will respond to the requests concerning the raw data and reasonable accommodations will be provided.

## Author Contributions

JW designed and simulated the IVT of virtual surgery and performed the data analysis. YY and XF provided support of clinical knowledge. The initial manuscript draft was prepared by JW and subsequently revised by AQ. All authors approved the final submitted version of the manuscript.

## Conflict of Interest

The authors declare that the research was conducted in the absence of any commercial or financial relationships that could be construed as a potential conflict of interest.
