# Corrigendum: Numerical Simulation of the Influence of Geometric Configurations on Pressure Difference in the Intraventricular Tunnel

**DOI:** 10.3389/fphys.2020.00256

**Published:** 2020-03-20

**Authors:** Yao Yang, Junjie Wang, Aike Qiao, Xiangming Fan

**Affiliations:** ^1^Beijing Anzhen Hospital, Capital Medical University, Beijing, China; ^2^College of Life Sciences and Bioengineering, Beijing University of Technology, Beijing, China

**Keywords:** double outlet right ventricle, intraventricular tunnel, hemodynamics, numerical simulation, surgical planning

In the original article, the author Xiangming Fan was marked as belonging to affiliation 2. The correct affiliation is 1. In addition, there was an error in the running title as it referred to “Flow Resistance.” The correct running title should be “Simulation of Pressure Difference in the Intraventricular Tunnel.”

The authors apologize for this error and state that this does not change the scientific conclusions of the article in any way. The original article has been updated.

